# Classifying nursing roles in critical infectious disease care: a multicenter cross-sectional study using cluster analysis

**DOI:** 10.3389/fpubh.2026.1792864

**Published:** 2026-03-20

**Authors:** Xiao Dang, Lijingzi Wang, Shuyao Wang, Pei Li, Wen Kang

**Affiliations:** Department of Infectious Diseases, Tangdu Hospital, Fourth Military Medical University, Xi'an, China

**Keywords:** cluster analysis, infectious diseases care, job content, nursing management, nursing roles

## Abstract

**Aim:**

To classify nursing roles in the care of critically ill patients with infectious diseases based on clinical practice patterns and nurse characteristics, and to describe the features of each role type to provide empirical evidence for optimising human resource allocation and enhancing care efficiency.

**Design:**

A multicenter cross-sectional survey study.

**Methods:**

Between February and March 2025, 275 nurses providing direct care to critically ill patients with infectious diseases were recruited from multiple tertiary hospitals across diverse geographic regions. Data were collected using a self-developed, semi-structured interview-informed questionnaire assessing the frequency of 80 specific nursing activities, alongside demographic and workload information. Hierarchical cluster analysis followed by K-means clustering was applied to identify latent nursing role types. The differences of each type were further compared by univariate analysis and multiple logistic regression analysis.

**Results:**

Three core domains of nursing practice were identified: clinical nursing care, advanced clinical specialty skills, and nursing management/professional development. Accordingly, three distinct nursing role types: Comprehensive Development Type —high engagement across all domains; Management and Coordination Type —primarily focused on administrative and coordination tasks with minimal direct patient care; and Clinical Task Execution Type—predominantly delivering routine basic care with limited involvement in advanced or managerial activities. These types differed significantly in age, professional experience, formal position, weekly workload, and intention to leave the role. Multiple regression analysis showed that traditional variables such as age and professional title had no significant independent predictive effect on distinguishing comprehensive development type from clinical task execution type; Age is the qualification threshold of management coordination.

**Conclusion:**

Nursing roles in critical infectious diseases care form an informal but functionally stratified hierarchy shaped by career progression and organisational demands, yet lack formal recognition. This empirically derived typology supports precision-based staffing, role clarification, and targeted retention strategies. Aligning job expectations with actual practice can reduce burnout—particularly among early-career nurses performing high-volume basic tasks—and strengthen team resilience during public health emergencies. Findings inform the development of flexible, evidence-based role frameworks that enhance both workforce sustainability and patient safety.

## 1 Introduction

In the context of frequent outbreaks of emerging and re-emerging infectious diseases globally, the care of critically ill patients with infectious diseases has become one of the core challenges confronting public health emergency response systems (1). These patients often present with multi-organ failure, high transmissibility, and rapidly deteriorating clinical conditions, requiring advanced life-support technologies such as mechanical ventilation, continuous renal replacement therapy, and extracorporeal membrane oxygenation. Nursing care in this setting is characterised by high intensity, elevated occupational risk, and a high degree of specialisation—commonly described in the literature as reflecting the “three highs” of critical infectious disease nursing (2, 3). In this high-stakes environment, nurses serve as pivotal members of frontline response teams, and the clarity, differentiation, and scientific allocation of their job responsibilities are directly linked to patient safety, care quality, and overall system efficiency.

However, current practice models for nursing roles in critical infectious disease care continue to face significant challenges related to role ambiguity and a lack of standardised responsibility frameworks. On one hand, staffing structures are often adapted from general infectious disease wards or conventional intensive care units, which may not adequately address the unique demands of this specialty—including stringent infection control protocols, psychological support under prolonged isolation, and dynamic coordination within multidisciplinary outbreak response teams (4, 5). On the other hand, task allocation frequently relies on experiential judgment rather than empirical evidence, resulting in suboptimal utilisation of professional competencies, mismatches between role expectations and actual practice, and heightened levels of occupational stress. These factors have been increasingly associated with burnout and intention to leave the profession, particularly among early-career nurses (6, 7).

In response, the concept of precision management has driven a paradigm shift in nursing workforce research—from “person-based” positions toward “responsibility-based” role design grounded in real-world practice data (8, 9). Within this framework, cluster analysis—an unsupervised machine learning technique—has emerged as a powerful tool for identifying latent subgroups based on behavioural patterns. It has been widely applied in healthcare for disease phenotyping, patient risk stratification, and clinician practice variation studies (10, 11). Recent nursing studies have begun to adopt this approach to delineate distinct role typologies in acute and critical care settings based on actual task performance. Nevertheless, its application specifically to the specialised domain of critical infectious disease nursing remains limited.

Based on Patricia Benner’s “from novice to expert” model, this study explored the functional differentiation of nurses’ role in infectious disease intensive care. Benner (12) pointed out that with the development of clinical experience and cognitive ability, nurses will experience five stages from novice, advanced beginner, competent, proficient to expert, and their clinical responsibilities, task complexity and professional influence will also evolve. This theory suggests that the combination of tasks performed by nurses in their daily work is the core external representation of their professional development stage and role orientation. Based on Benner’s theory and combined with the empirical research related to the hierarchical management of specialist nurses under this theory (13), this study puts forward the first hypothesis: the participation mode of infectious disease intensive care nurses in clinical nursing, nursing management and professional technology tasks can be clustered into several role types that can be distinguished empirically and reflect the level of ability.

In addition, this study draws on the work demand resource model. This model was proposed by Demerouti et al. (14) and later improved by Bakker and demerouti (15). It emphasizes that the specific configuration of work characteristics (needs and resources) shapes employees’ work behavior, experience and role orientation. In the context of nursing organization, demographic characteristics and professional attributes (such as age, professional title, position, workload) reflect the work demand intensity and available work resources level faced by nurses to a certain extent. Relevant empirical studies also show that the above factors have an important impact on the role differentiation and ability development of nurses (16, 17). Therefore, we further propose the second hypothesis: the role types generated by the above clustering are related to key demographic characteristics, occupational attributes and workload indicators (such as age, professional title, position, working hours, overtime frequency). This reflects the working mode in which the role structure is shaped by personal ability development and system needs in the high-risk nursing environment.

Therefore, this study focuses on nurses directly engaged in the care of critically ill patients with infectious diseases. Using a multicenter cross-sectional design, we collected data on the frequency of 80 specific nursing activities performed during routine practice. Applying K-means cluster analysis, we aim to: (1) identify empirically derived typologies of nursing roles based on real-world responsibility patterns in critical infectious disease care; (2) describe how these typologies differ in terms of demographic characteristics, formal positions, workload, and retention-related indicators; and (3) provide actionable evidence to support the development of a differentiated, competency-aligned, and evidence-based job responsibility framework—ultimately informing precise human resource allocation and sustainable workforce strategies in high-risk infectious disease settings.

## 2 Methods

### 2.1 Design

This study employed a cross-sectional survey design and was conducted via an anonymous online questionnaire between February and March 2025.

### 2.2 Reporting transparency

The manuscript was prepared in accordance with the Strengthening the Reporting of Observational Studies in Epidemiology (STROBE) statement guidelines for observational studies.

### 2.3 Study setting and sampling

A total of 275 nurses providing care for critically ill patients with infectious diseases were recruited from healthcare institutions across seven provinces and municipalities. Participants were recruited through the social media platform and completed the survey online. Based on factor analysis requirements—typically recommending a sample size at least 10 times the number of variables—the study considered 11 demographic variables and anticipated 3–5 latent job responsibility categories, resulting in 14–19 analytical variables. Accounting for an estimated 20% invalid response rate, the required sample size was calculated to be between 175 and 238 participants.

### 2.4 Inclusion and exclusion criteria

Inclusion criteria were: (1) currently employed as a clinical nurse; (2) working in a unit that admits critically ill patients with infectious diseases (e.g., ICU, infectious disease intensive care unit, or wards with designated critical care beds); (3) directly or indirectly involved in the nursing care of such patients; and (4) providing informed consent to participate.

Exclusion criteria included: (1) nurses not actively employed at the participating hospitals during the survey period; and (2) non-permanent staff such as trainees or student nurses.

### 2.5 Survey instrument

A self-developed instrument, the Survey on Content of nursing practice for critically ill patients with infectious diseases, was used. The questionnaire was developed based on clinical needs, supported by literature review and semi-structured interviews with nurses of varying experience levels. Initial items were refined following feedback from infectious disease physicians and nursing experts to address ambiguities, unclear phrasing, or clinical irrelevance.

The final instrument demonstrated excellent internal consistency, with a Cronbach’s *α* coefficient of 0.965. It comprised two sections: (1) Demographic and professional characteristics, including age, education level, professional title, and years of clinical experience; (2) Content of nursing practice, consisting of 80 items across multiple domains—basic nursing care, technical procedures, research/management, and emergency response. Respondents rated the frequency of performing each task on a 6-point Likert scale:1 = Not performed (task done by physicians, nursing assistants, or others), 2 = Very rarely (0 times per week / almost never), 3 = Occasionally (1–2 times per week), 4 = Sometimes (3–4 times per week / moderate frequency), 5 = Often (5–6 times per week / nearly daily), 6 = Very frequently (7 + times per week / multiple times daily).

### 2.6 Data collection

Data were collected online. To ensure anonymity and confidentiality, no personally identifiable information was collected. Quality control measures included: (1) mandatory responses for all items—participants received prompts to complete missing answers before submission;

(2) scrutiny of responses completed in less than 5 min or showing repetitive patterns. A total of 294 questionnaires were collected. After dual independent review, 19 were excluded due to insufficient completion time (<5 min) or obvious response regularity, yielding 275 valid responses (effective response rate: 93.5%).

### 2.7 Data analysis

Data were analyzed using IBM SPSS Statistics version 25.

Descriptive statistics and cluster analyses were performed. To mitigate the influence of differing scales across the 80 job responsibility items, Z-score standardization was applied prior to factor and cluster analyses.

Hierarchical clustering (using average linkage between groups and squared Euclidean distance) was first conducted on the 80 standardized variables to explore natural groupings among job tasks. Subsequently, K-means clustering was employed to identify the optimal number of distinct nurse responsibility profiles. The number of clusters (k) was evaluated for k = 2 to k = 5 by examining the within-cluster sum of squares (WCSS); the “elbow method” was used to select the k value at which the rate of decrease in WCSS markedly slowed.

The resulting cluster membership served as the grouping variable for comparative analyses. Cross-tabulations with chi-square tests (or Fisher’s exact test where appropriate) were used to examine differences across clusters in demographic and professional characteristics, thereby validating the meaningfulness and discriminant validity of the identified typologies.

Univariate analysis: chi square test or Fisher’s exact test was used to examine the differences in demographic and occupational characteristics (age, title, registered nurse status, head nurse status, average overtime hours) among three types of nurses. The results provide a basis for variable selection in multivariate analysis.

Multiple logistic regression: The dependent variables are three types of nurse roles. Two separate models were installed using C-type and A-type as reference categories to fully distinguish each character pair. Evaluate the model using likelihood ratio test, Nagelkerke pseudo R^2^, Pearson and bias chi square test. The magnitude of the effect is represented by odds ratio (OR) and 95% confidence interval. Due to the fact that polynomial logistic regression does not output collinearity diagnosis, auxiliary linear regression was performed on the predictive factors of the virtual encoding of the independent variables. The range of all VIFs in this study is 1.02–5.35 (<10), indicating no severe multicollinearity.

### 2.8 Ethical considerations

The authors affirm that all procedures in this study complied with national and institutional ethical standards for human research and adhered to the principles of the Declaration of Helsinki (as revised in 2008). The study was approved by the Institutional Ethics Committee (Approval No.: ZHHLXH-Y-20250-02). Potential participants received a digital invitation via the online questionnaire, which detailed the study purpose and their rights, including voluntary participation and the right to withdraw. Completion and submission of the questionnaire were regarded as implied informed consent.

## 3 Results

### 3.1 Construction of a questionnaire on nursing practice for critically ill patients with infectious diseases

Through a literature review and semi-structured interviews with nurses of various experience levels, an initial draft of the survey questionnaire was developed, containing 89 items. After review and revision by a panel of physicians and nursing experts, the final nursing practice content for critically ill patients with infectious diseases consisted of 80 items (see Table 1). This checklist focuses on practical work content relevant to the care of critically ill infectious disease patients, encompassing basic nursing care, specialized technical operations, and research/management coordination.

**Table 1:** Nursing practice content for critically ill patients with infectious diseases.

No.	Item
1	Participate in shift handover to understand patient flow and dynamic changes in clinical condition
2	Inquire about and document patients’ epidemiological history, allergy history, past medical history, etc., upon admission
3	Promptly implement necessary measures (e.g., strict or protective isolation) for newly admitted high-risk patients or inpatients with high transmissibility
4	Guide patients and their families on isolation protocols and home-based protective measures
5	Provide admission, discharge, and various types of health education (e.g., visitation/companionship policies, safety hazards)
6	Stay informed about the treatment plan for critically ill patients and communicate with attending physicians to adjust critical care plans and nursing interventions
7	Conduct electronic risk assessments (e.g., self-care ability, pressure injury, catheter dislodgement) and implement corresponding interventions
8	Perform intensive monitoring (e.g., vital signs, pupil and consciousness status, fluid intake/output, electrolyte balance, cardiac output, intra-abdominal pressure)
9	Administer medications via oral, nasogastric, or nasoenteric routes
10	Observe and manage common adverse drug reactions in infectious disease patients
11	Provide tailored dietary guidance based on the type of infectious disease
12	Assist patients with eating
13	Perform nasogastric feeding care and assess/monitor enteral nutrition status
14	Manage complications related to enteral nutrition
15	Perform intravenous infusion, blood transfusion, intramuscular or subcutaneous injections
16	Administer and maintain advanced vascular access devices (e.g., PICC, CVC, implanted ports)
17	Insert and secure tubes/catheters (e.g., nasogastric tube, urinary catheter, arterial/venous lines, thoracic/abdominal drains) and monitor patient responses
18	Maintain tubes/catheters and manage associated adverse events
19	Perform oxygen therapy, nebulization, suctioning, mechanical assisted expectoration, and pneumatic compression therapy
20	Perform enemas, skin care, rectal tube decompression, oral care, urethral orifice care, and perineal irrigation
21	Collect and promptly deliver various specimens (e.g., venous/arterial blood, blood glucose, sputum, urine, stool)
22	Provide activities of daily living (ADL) care to maintain “three trims and six cleans” (a Chinese nursing standard for personal hygiene)
23	Observe skin for rashes, lesions, petechiae, etc.
24	Perform wound dressing changes (large, medium, small), blister fluid aspiration, and debridement of skin ulcers
25	Implement positioning strategies to prevent complications such as pressure injuries
26	Provide individualized physical cooling for febrile patients
27	Conduct limb and respiratory function training
28	Assist patients with rehabilitation exercises
29	Identify psychological distress in patients and families and provide psychosocial support
30	Prepare bed units and necessary supplies before patient admission
31	Handle excreta and contaminated linens from infectious patients according to protocol
32	Perform terminal disinfection of bed units and rooms after patient discharge
33	Perform postmortem care
34	Comprehensively analyze actual or potential nursing problems in critically ill infectious disease patients and implement targeted interventions
35	Provide nursing support during common infectious disease procedures (e.g., liver biopsy, paracentesis, hemodialysis, ascites concentration, plasma exchange, lumbar puncture, Sengstaken–Blakemore tube placement)
36	Participate in resuscitation of critically ill patients, including CPR, defibrillation, and administration of emergency medications
37	Organize and coordinate nursing activities during resuscitation
38	Rapidly identify and manage adverse reactions to emergency medications
39	Interpret laboratory results and manage critical values in infectious disease patients
40	Participate in multidisciplinary team collaboration for complex infectious disease cases
41	Deliver end-of-life care for critically ill infectious disease patients
42	Inspect emergency equipment, supplies, and controlled/high-alert/expensive medications daily; ensure shift-to-shift handover documentation
43	Perform environmental disinfection of surfaces, air, and designated areas
44	Inspect and ensure proper functioning of medical equipment
45	Maintain and disinfect medical equipment
46	Monitor ward temperature, humidity, and negative-pressure room operation
47	Patrol internal and external ward environments and emergency access routes
48	Participate in emergency response drills
49	Calculate consumable charges, bill patients appropriately, print daily expense statements, and provide explanations
50	Coordinate with other departments (e.g., laboratory, radiology) to facilitate timely patient examinations
51	Process standing and STAT physician orders; prepare corresponding medications
52	Manage medical waste according to regulations
53	Accurately and timely document nursing records
54	Operate and maintain basic life-support equipment (e.g., invasive/non-invasive ventilators, high-flow oxygen therapy devices, invasive arterial blood pressure monitors, enteral feeding pumps)
55	Operate and maintain advanced life-support and monitoring equipment (e.g., cardiac output monitors, PiCCO, ECMO)
56	Provide disease-specific health consultations and participate in patient follow-up management
57	Participate in developing or revising departmental policies and workflows
58	Supervise, guide, and review nursing documentation and tasks performed by junior staff
59	Monitor and improve nursing quality among junior staff; participate in continuous quality improvement initiatives
60	Manage inventory and coordinate emergency supplies; inspect expiration dates and quantities of routine items, PPE, and emergency stock
61	Audit compliance with infection prevention and control measures in the ward
62	Conduct quality control of inpatient and discharged nursing records to ensure documentation standards
63	Create flexible staffing schedules and reallocate human resources during emergencies
64	Arrange periodic environmental air cultures and hand swab tests for healthcare workers
65	Report safety incidents involving junior nurses, trainees, and interns
66	Assist senior nurses and head nurses in providing occupational protection guidance and verifying post-exposure protocols
67	Revise nurse performance evaluation criteria
68	Coordinate personnel deployment and workflow during public health emergencies
69	Report patient-related adverse events in the ward
70	Train nursing assistants on safety protocols and care skills
71	Provide training and assessment on infectious disease prevention and nursing competencies for staff nurses
72	Deliver theoretical instruction, skills training, and evaluations for student nurses; supervise case presentations, nursing rounds, and thesis writing
73	Develop teaching materials and lesson plans; provide theoretical instruction and skill training for new hires, junior nurses, and student nurses
74	Develop teaching materials and lesson plans; provide advanced theoretical instruction and skill training for visiting and specialty nurses
75	Implement new technologies or clinical innovations
76	Apply for research grants, awards, patents, or software copyrights
77	Participate in scientific innovation competitions and clinical skills contests
78	Publish articles in academic journals or conference proceedings
79	Write clinical nursing case reports
80	Contribute to the development of public health education and science communication materials

### 3.2 Clustering results of nursing practice content for critically ill patients with infectious diseases

**Figure 1:**
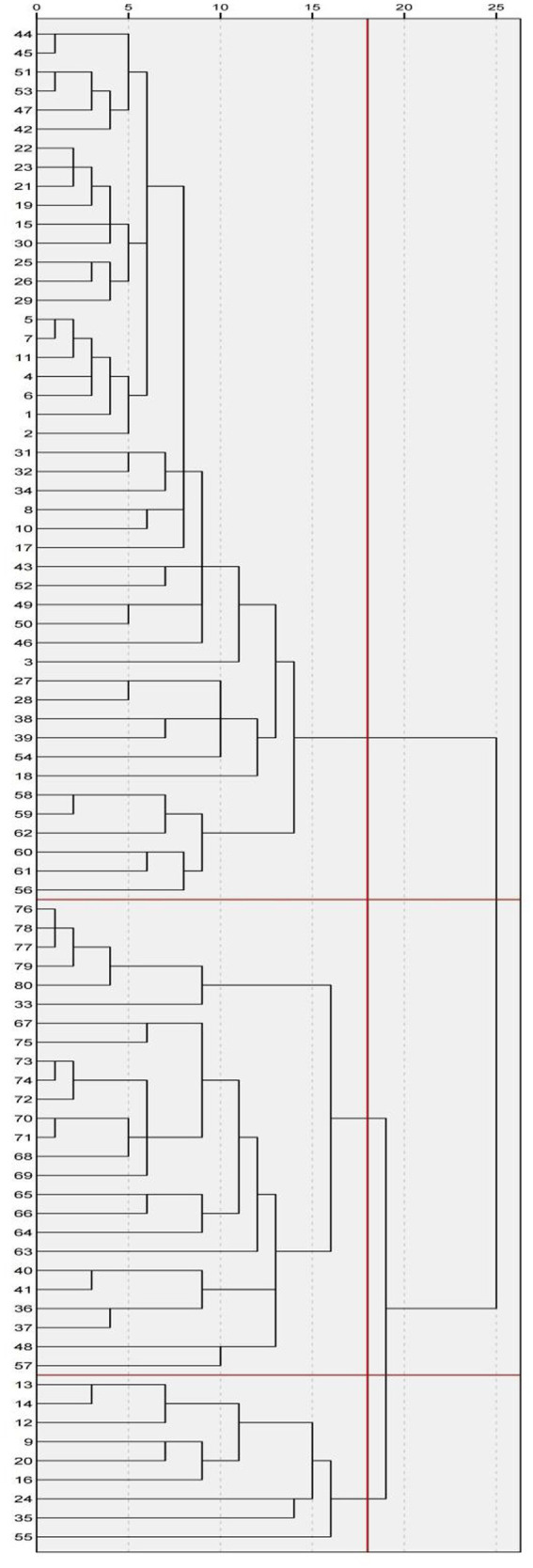
Spectral analysis of nursing practice content for critically ill patients with infectious diseases.

Hierarchical clustering analysis was performed on the 80 job responsibility items based on clinical relevance. As shown in Figure 1, when the inter-group distance x = 18, the nursing roles in critical infectious disease care were categorized into three clusters:

Cluster I: Comprises 47 items (items 1–8, 10–11, 15, 17–19, 21–23, 25–32, 34, 38–39, 42–47, 49–54, 56, 58–62), mainly covering comprehensive clinical nursing practices for critically ill infectious disease patients, including patient assessment, planning and implementation of care (e.g., basic care, therapeutic procedures, medication management, health education), evaluation of outcomes (monitoring vital signs and dynamic changes in condition), and foundational management tasks essential for ensuring safe operation of isolation wards (e.g., infection control measures, equipment maintenance, documentation, cost management).

Cluster II: Includes 24 items (items 33, 36–37, 40–41, 48, 57, 63–80), focusing on management, coordination, and professional development within the nursing team, such as human resource allocation, scheduling, performance evaluations, policy revisions, organizing emergency drills, handling adverse events, providing teaching and training for on-duty and continuing education nurses, participating in research activities, and promoting discipline construction.

Cluster III: Consists of 9 items (items 9, 12–14, 16, 20, 24, 35, 55), involving advanced specialized skills applied in the care of critically ill infectious disease patients, such as managing enteral nutrition complications, maintaining advanced vascular access (PICC, CVC, infusion ports), complex wound management, performing various punctures and invasive procedures like ECMO, and operating and managing high-end life support and monitoring equipment.

Based on these characteristics, the three clusters were named: Clinical Nursing Practice, Nursing Management and Professional Development, and Advanced Clinical Specialized Techniques (Figure 1).

### 3.3 Clustering analysis results of nursing roles in critical infectious disease care

**Figure 2:**
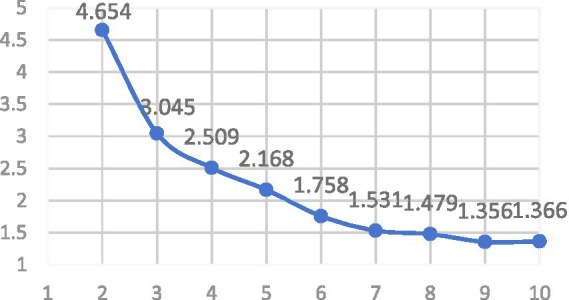
K-means clustering method “elbow” analysis results.

**Figure 3:**
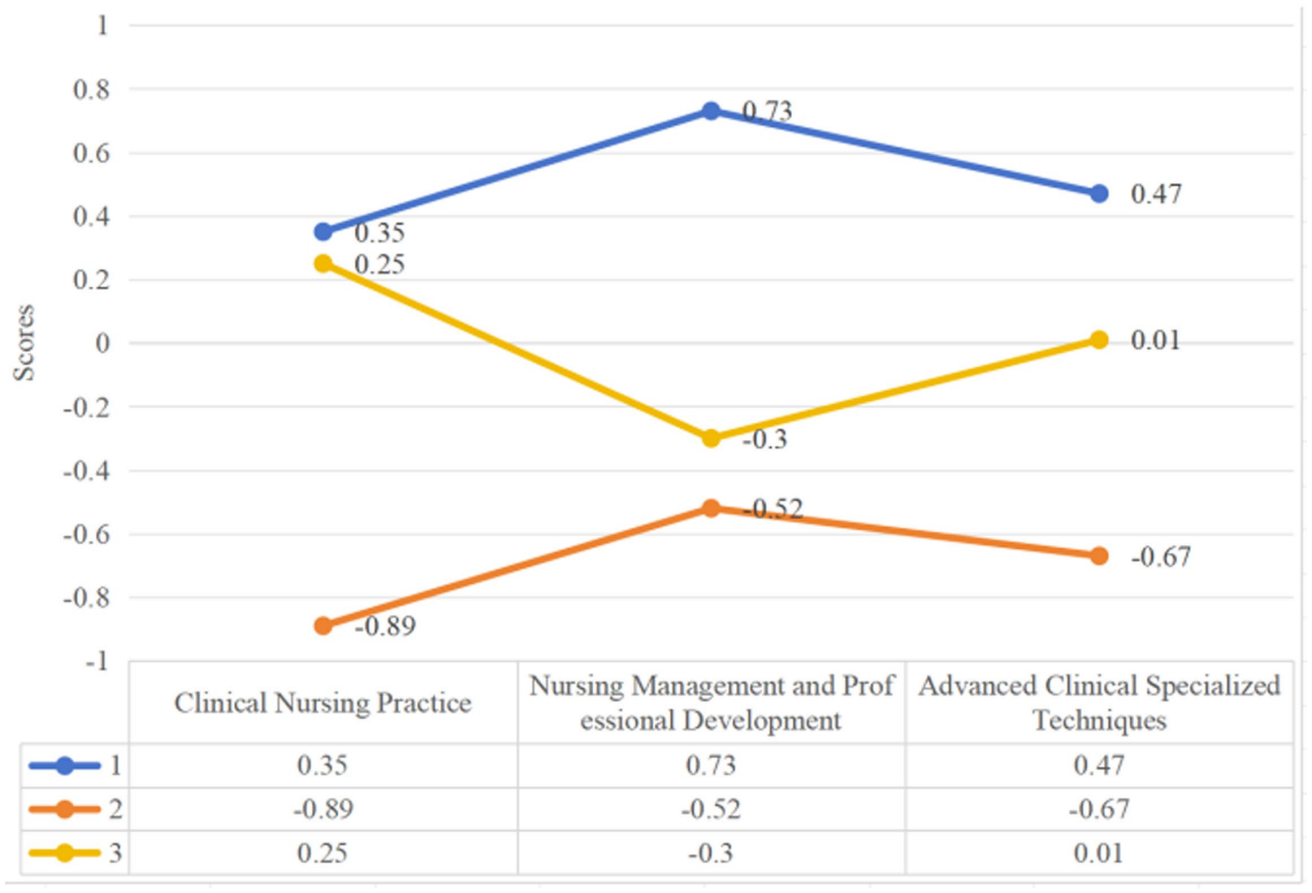
The role types of nurses in critically ill patients with infectious diseases based on K-means clustering analysis.

K-Means clustering analysis was conducted on the frequency data of job responsibilities from 275 nurses. The optimal number of clusters was determined using the Elbow Method, taking into account the inflection point of the curve, interpretability of clusters, and expert recommendations, ultimately settling on K = 3 as the optimal typology solution (Figure 2). The characteristics of the three typologies are as follows: Comprehensive Development Type(Type A): High scores across Clinical Nursing Practice, Nursing Management and Professional Development, and Advanced Clinical Specialized Techniques. Core responsibilities include comprehensive care for critically ill patients, quality control management, teaching guidance, and participation in research. Management Coordination Type(Type B): Relatively higher scores in Nursing Management and Professional Development but lower involvement in Clinical Nursing and Advanced Techniques. Main roles involve team coordination, human resource scheduling, emergency response, and administrative tasks. Clinical Task Execution Type (TypeC): Highest scores in Clinical Nursing Practice but minimal involvement in Management and Advanced Techniques. Core responsibilities focus on daily basic care, routine treatment execution, and simple procedures for critically ill patients (Figure 3).

### 3.4 Differences in personnel characteristics across different nursing roles

The age range of the 275 nurses was 20–54 years old (mean ± SD: 35.01 ± 6.998). Significant differences (*p* < 0.05) were observed across the different role types in terms of age, position, professional title, overtime work, and other demographic characteristics. Additionally, significant differences (*p* < 0.05) were found in the frequency scores across the three dimensions—Clinical Nursing Practice, Nursing Management and Professional Development, and Advanced Clinical Specialized Techniques. Detailed pairwise comparisons are presented in Tables 2, 3.

**Table 2:** Socio-demographic distribution characteristics of nurses caring for critically ill patients with infectious diseases by different role types.

Item	Category	Total	Type A	Type B	Type C	Statistic	*p*-value
		(*n* = 275) %	(*n* = 95)	(*n* = 69)	(*n* = 111)		
Gender	Male	12 (4.4%)	4 (4.2%)	3 (4.3%)	5 (4.5%)	0.011	0.995
	Female	263 (95.6%)	91 (95.8%)	66 (95.7%)	106 (95.5%)		
Age(year)	<25	23 (8.4%)	6 (6.3%)	5 (7.2%)	12 (10.8%)	18.966	0.004
	26–34	104 (37.8%)	36 (37.8%)	19 (27.5%)	49 (44.1%)		
	35–45	121 (44.0%)	46 (48.4%)	30 (43.5%)	45 (40.5%)		
	>45	27 (9.8%)	7 (7.4%)	15 (21.7%)	5 (4.5%)		
Education level	Associate degree	20 (7.3%)	6 (6.3%)	9 (13.0%)	5 (4.5%)	4.830	0.305
	Bachelor’s degree	246 (89.5%)	86 (90.5%)	58 (84.1%)	102 (91.9%)		
	Master’s degree	9 (3.3%)	3 (3.2%)	2 (2.9%)	4 (3.6%)		
Work experience	≤6 Months	4 (1.5%)	3 (3.2%)	1 (1.4%)	0 (0%)	5.500	0.240
	6 Months–1 Year	17 (6.2%)	6 (6.3%)	2 (2.9%)	9 (8.1%)		
	>1 Year	254 (92.4%)	86 (90.5%)	66 (95.7%)	102 (91.9%)		
Position/role (multiple choices)	Registered nurse	212 (77.1%)	65 (68.4%)	58 (84.1%)	89 (80.2%)	6.540	0.038
	Quality control nurse	103 (37.5%)	33 (34.7%)	27 (39.1%)	43 (38.7%)	0.460	0.794
	Preceptor Nurse	105 (38.2%)	39 (41.1%)	26 (37.7%)	40 (36.0%)	0.556	0.757
	Specialty nurse	113 (41.1%)	35 (36.8%)	26 (37.7%)	52 (46.8%)	2.559	0.278
	Community nurse	2 (0.7%)	1 (1.1%)	0 (0%)	1 (0.9%)	0.691	0.708
	Head nurse	49 (17.8%)	20 (21.1%)	18 (26.1%)	11 (9.9%)	8.641	0.013
	Research nurse	10 (3.6%)	4 (4.2%)	3 (4.3%)	3 (2.7%)	0.465	0.792
Professional title	Junior	112 (40.7%)	41 (43.2%)	20 (29.0%)	51 (45.9%)	11.040	0.026
	Intermediate	134 (48.7%)	41 (43.2%)	38 (55.1%)	55 (49.5%)		
	Senior	29 (10.5%)	13 (13.7%)	11 (15.9%)	5 (4.5%)		
Hospital type	Public hospital	255 (92.7%)	86 (90.5%)	63 (91.3%)	106 (95.5%)	2.151	0.341
	Private hospital	20 (7.3%)	9 (9.5%)	6 (8.7%)	5 (4.5%)		
Hospital level	Tertiary hospital	224 (81.5%)	78 (82.1%)	53 (76.8%)	93 (83.8%)	1.410	0.494
	Non-tertiary hospital	51 (18.5%)	17 (17.9%)	16 (23.2%)	18 (16.2%)		
Average overtime hours in the past 3 months (excluding morning shift handover)	No overtime	18 (6.5%)	10 (10.5%)	3 (4.3%)	5 (4.5%)	18.766	0.016
	0.5–1 h	122 (44.4%)	37 (38.9%)	44 (63.8%)	41 (36.9%)		
	1–2 h	78 (28.4%)	27 (28.4%)	15 (21.7%)	36 (32.4%)		
	2–4 h	48 (17.5%)	17 (17.9%)	6 (8.7%)	25 (22.5%)		
	≥4 h	9 (3.3%)	4 (4.2%)	1 (1.4%)	4 (3.6%)		
Usual weekly working hours	≤40 h	65 (23.6%)	28 (29.5%)	17 (24.6%)	20 (18.0%)	3.773	0.152
	>40 h	210 (76.4%)	67 (70.5%)	52 (75.4%)	91 (82.0%)		
Intention to leave	Yes	58 (21.1%)	23 (24.2%)	13 (18.8%)	22 (19.8%)	0.873	0.646
	No	217 (78.9%)	72 (75.8%)	56 (81.2%)	89 (80.2%)		

**Table 3:** Comparison of scores on three categories of nursing tasks among nurses caring for critically ill patients with infectious diseases by different role types (mean ± SD).

Nursing task	Type A	Type B	Type C	*F*-value	*p*-value	Pairwise comparison
Basic clinical nursing practice	0.35 ± 0.41	−0.89 ± 0.52	0.25 ± 0.30	224.365	<0.001	1 > 2, 3 > 2
Nursing research and management	0.73 ± 0.45	−0.51 ± 0.40	−0.30 ± 0.38	238.045	<0.001	1 > 3 > 2
Advanced clinical specialized techniques	0.47 ± 0.50	−0.67 ± 0.34	0.01 ± 0.49	122.898	<0.001	1 > 3 > 2

### 3.5 Multiple logistic regression analysis of demography and occupational characteristics in different nurse role classification

Three types of nurse roles obtained through cluster analysis Type A: comprehensive developmental type; Type B: Management and Coordination Type; Two multinomial logistic regression models were constructed with clinical task execution type (C type) and comprehensive developmental type (A type) as reference categories, with clinical task execution type (C type) as the dependent variable. The overall fitting information of the two models is consistent: the final model likelihood ratio test is chi squared = 52.887, df = 22, *p* < 0.001, Nagelkerke pseudo R^2^ = 0.198, indicating that the model has statistical significance and can explain 19.8% of the variation in nurse role classification. The goodness of fit test showed Pearson *χ*^2^ = 136.036 (df = 120, *p* = 0.150), bias *χ*^2^ = 132.891(df = 120, *p* = 0.199). None of them showed statistical significance, indicating good model calibration.

#### 3.5.1 Referring to clinical task execution type (type C)

##### 3.5.1.1 Comprehensive development type vs. clinical task execution type

As shown in Table 4, after controlling for other variables, there was no statistically significant difference in the levels of age, professional title, registered nurse status, head nurse status, and average overtime hours between Type A and Type C (all *p* > 0.05).

**Table 4:** Multiple logistic regression results of role classification of infectious disease intensive care nurses.

Comparison of nurse role types	Variable	*B*	*SE*	Wald	*p*	OR	95%CI	
							Lower	Upper
TypeA vs. TypeC(based on Type C)	Constant	0.808	1.047	0.595	0.44			
	Age (<25 years)	−0.826	0.906	0.831	0.362	0.438	0.074	2.585
	Age (26–34 years)	−0.232	0.748	0.096	0.757	0.793	0.183	3.438
	Age (35–45 years)	0.103	0.669	0.023	0.878	1.108	0.298	4.115
	Only registered nurse (NO)	0.605	0.363	2.78	0.095	1.831	0.899	3.728
	Head nurse (NO)	−0.461	0.492	0.877	0.349	0.631	0.24	1.655
	Professional title (Junior)	−0.428	0.702	0.372	0.542	0.652	0.165	2.58
	Professional title (Intermediate)	−1.034	0.612	2.853	0.091	0.355	0.107	1.18
	No overtime	0.935	0.947	0.976	0.323	2.547	0.398	16.282
	Average overtime hours (0.5–1 h)	0.189	0.79	0.057	0.811	1.208	0.257	5.684
	Average overtime hours (1–2 h)	−0.033	0.795	0.002	0.967	0.968	0.204	4.594
	Average Overtime Hours(2–4 h)	−0.283	0.832	0.115	0.734	0.754	0.148	3.85
TypeB vs. TypC (based on Type C)	Constant	0.981	1.354	0.525	0.469			
	Age (<25 years)	−1.701	0.908	3.507	0.061	0.182	0.031	1.083
	Age (26–34 years)	−1.452	0.712	4.157	0.041	0.234	0.058	0.945
	Age (35–45 years)	−1.429	0.611	5.464	0.019	0.24	0.072	0.794
	Only registered nurse (NO)	−0.637	0.48	1.761	0.185	0.529	0.206	1.355
	Only head nurse (Reference: YES)							
	Head nurse (NO)	−0.921	0.539	2.916	0.088	0.398	0.138	1.146
	Professional title (Reference: Senior)							
	Professional title (junior)	−0.717	0.794	0.815	0.367	0.488	0.103	2.315
	Professional title (intermediate)	−0.489	0.662	0.544	0.461	0.613	0.167	2.247
	Average overtime hours (no overtime)	1.177	1.388	0.719	0.397	3.244	0.214	49.27
	Average overtime hours (0.5–1 h)	1.819	1.191	2.336	0.126	6.168	0.598	63.607
	Average overtime hours (1–2 h)	0.805	1.21	0.443	0.506	2.237	0.209	23.971
	Average overtime hours (2–4 h)	0.407	1.265	0.103	0.748	1.502	0.126	17.932
TypeA vs. TypeB (based on Type A)	Constant	0.174	1.272	0.019	0.891			
	Age (<25 years)	−0.875	0.954	0.84	0.359	0.417	0.064	2.707
	Age (26–34 years)	−1.22	0.723	2.85	0.091	0.295	0.072	1.217
	Age (35–45 years)	−1.532	0.585	6.865	0.009	0.216	0.069	0.68
	Only registered nurse (NO)	−1.242	0.467	7.066	0.008	0.289	0.116	0.722
	Head nurse (NO)	−0.46	0.506	0.827	0.363	0.631	0.234	1.701
	Professional title (Junior)	−0.289	0.731	0.156	0.693	0.749	0.179	3.137
	Professional title (Intermediate)	0.546	0.569	0.92	0.338	1.726	0.566	5.262
	Average overtime hours (no overtime)	0.242	1.328	0.033	0.856	1.274	0.094	17.204
	Average overtime hours (0.5–1 h)	1.63	1.174	1.93	0.165	5.106	0.512	50.93
	Average Overtime hours (1–2 h)	0.838	1.197	0.49	0.484	2.312	0.221	24.133
	Average overtime hours (2–4 h)	0.689	1.256	0.301	0.583	1.992	0.17	23.357

All independent variables were categorical and entered into the models as factors. The specific coding and reference group are as follows: 1, <25 years; 2, 26–34 years; 3, 35–45 years; 4 = > 45 years (reference); Professional title: 1, Junior; 2, Intermediate; 3, Senior (reference); Registered nurse-only status: 0, No (holds additional responsibilities); 1, Yes (reference); Head nurse status: 0, No; 1, Yes (reference); Average daily overtime hours: 1, No overtime; 2, 0.5–1 h; 3, 1–2 h; 4, 2–4 h; 5, ≥4 h (reference).

#### 3.5.1.2 Management coordination type vs. clinical task execution type

Age is the only statistically significant independent predictor. Compared to nurses over 45 years old, nurses aged 26–34 years old who were classified as type B had a significantly lower advantage (OR = 0.234, 95% CI: 0.058–0.945, *p* = 0.041). In other words, the advantage of classifying nurses over 45 years old as type B is 4.27 (1/0.234) times that of nurses aged 26–34. Nurses aged 35–45 have a significantly lower advantage in being classified as type B (OR = 0.240, 95% CI: 0.072–0.794, *p* = 0.019), meaning that nurses over 45 years old have a 4.17-fold advantage in being classified as type B compared to nurses aged 35–45. The difference between nurses under the age of 25 and nurses over the age of 45 did not reach statistical significance (OR = 0.182, 95% CI: 0.031–1.083, *p* = 0.061), but showed a similar trend. Other variables, including professional title, registered nurse status, head nurse status, and average overtime hours, did not show statistical significance at all levels (all *p* > 0.05).

#### 3.5.2 Taking comprehensive development type (type a) as a reference

##### 3.5.2.1 Management coordination type vs. comprehensive development type

Age and registered nurse status are significant independent predictive factors. Compared to type A, nurses aged 35–45 who were classified as type B had a significantly lower advantage (OR = 0.216, 95% CI: 0.069–0.680, *p* = 0.009) when compared to those over 45 years old. In other words, the advantage of classifying nurses over 45 years old as type A is 4.63 times that of nurses aged 35–45 years old. The difference between nurses under 25 years old (OR = 0.417, 95% CI: 0.064–2.707, *p* = 0.359) and nurses over 45 years old (OR = 0.295, 95% CI: 0.072–1.217, *p* = 0.091) was not statistically significant.

Registered nurse status: Referring to “only registered nurses (yes)”, the advantage of being classified as type B (relative to type A) for non registered nurses (who also have other responsibilities) is significantly lower (OR = 0.289, 95% CI: 0.116–0.722, *p* = 0.008). In other words, the advantage of being classified as Type A for nurses who also have other responsibilities is 3.46 times that of only registered nurses. Other variables, such as professional title, head nurse status, and average overtime hours, did not show statistical significance at all levels (all *p* > 0.05). The specific parameters are shown in Table 4.

## 4 Discussion

### 4.1 Cluster the roles of nurses in three categories of infectious disease intensive care based on specific practical activities

This study identified three distinct nursing role types among 275 nurses providing direct care to critically ill patients with infectious diseases, using a multicenter cross-sectional design and K-means cluster analysis: the Comprehensive Development Type, the Management and Coordination Type, and the Clinical Task Execution Type. This result directly verifies our research hypothesis that the participation mode of nurses in clinical nursing, nursing management and professional technical tasks can be clustered into the role types that can be differentiated empirically and reflect the ability level.

The task combination of the three roles shows a gradient feature from basic execution to management coordination, and then to the combination of clinical, management and teaching, which is consistent with Benner’s theory that the scope of responsibility expands with the development of ability in the five stage model of “from novice to expert”. More importantly, these role types are not derived from any formal institutional definition, but from the functional stratification that naturally emerges in daily practice. This highlights the value of nurse role recognition based on actual data, which captures the real working state that cannot be recorded in the traditional personnel files.

### 4.2 Practical connotation of three types of nurses’ roles

Comprehensive Development Type (Type A, *n* = 95, 34.5%).

Nurses in this group demonstrated high engagement across all three practice domains, with particularly elevated involvement in nursing management and professional development activities (Z score = 0.73), alongside moderate participation in both basic (Z score = 0.35) and advanced clinical care (Z score = 0.47). They were predominantly mid-career professionals (48.4% aged 35–45 years) holding roles such as preceptor (41.1%), quality control nurse (34.7%), or head nurse (21.1%). Many cared for patients requiring complex interventions, including ventilator support or hemodialysis (16.8%). Clinically, this group plays a key role in integrating evidence into practice, guiding junior staff and coordinating the complex care of critically ill patients. Their multiple concerns in management, teaching and patient quality control are partly consistent with the role model of international senior practice nurses or clinical specialist nurses (18). However, although type a nurses have high comprehensive ability, they lack formal advanced practice certification, leading to their influence relying on the informal authority formed by experience accumulation. This may be a common problem in the health system of middle-income countries. Due to the lack of institutional support, high-capacity nurses are forced to expand their functions in the institutional gap (19).

As the operational core of critical infectious disease units, these nurses embody the proficient/expert stage in Benner’s theory. The JD-R model suggests that while they face high job demands from multiple responsibilities, they also possess substantial job resources—enabling sustained high engagement and performance. Nevertheless, this multifaceted workload is associated with elevated overtime and the highest turnover intention (24.2%) among all groups. This may be attributed to China’s performance appraisal system, which emphasizes clinical output over professional development, leaving insufficient protected time for research, teaching, and managerial duties (20, 21). To address this, we recommend that 20–30% of weekly working hours be institutionally protected for teaching, quality improvement, and policy revision (22). Improving the nursing practice environment and establishing competency-based certification and promotion pathways are also critical to sustaining this workforce.

Management and Coordination Type (Type B, *n* = 69, 25.1%).

This group exhibited the lowest engagement in direct clinical care (Z score = −0.89) and advanced specialty skills (Z score = −0.67), reflecting a primary focus on administrative and coordination functions. A higher proportion held senior positions, including the largest share of head nurses (26.1%), and many had extensive professional experience (21.7% > 45 years). This classification represents a vertical transformation of functions, from direct caregivers to system operation and maintenance personnel. This transformation is usually accompanied by formal organizational authorization, rather than the natural extension of clinical capabilities. From the perspective of JD-R model, the type of work needs has changed qualitatively (from physical/emotional needs to cognitive/coordination needs), and the work resources (position power, information control) have also been reconstructed. In this study, the average daily overtime length of type B group>2 h was the lowest among the three types of nurses, accounting for 10.1%, and their turnover intention was the lowest, which may be related to the nursing environment in which part of the group were promoted to nursing managers or reduced infectious diseases (23).

However, there are still some type B nurses who do not play the role of nursing manager, but assume administrative responsibilities. Although he seldom participates in clinical practice, he is often pulled back to the clinical front line due to the shortage of clinical manpower, and is still undertaking some clinical nursing work, which leads to the weakening of the strategic function of this role. Chen et al. (24) shows that if managers need to take into account the clinical, their leadership effectiveness will decline. Therefore, for type B nurses, it is suggested to improve their coordination ability through targeted training of emergency leadership for critical infectious diseases, increase full-time nursing management posts or nursing management auxiliary posts, and implement non clinical performance assessment with team effectiveness as the core, so as to avoid leadership exhaustion caused by insufficient manpower.

Clinical Task Execution Type (Type C,n = 111, 40.3%).

As the largest group, these nurses concentrated on routine bedside care, basic treatments, and simple procedures. They scored highest in basic clinical tasks (Z score = 0.25) but showed minimal involvement in advanced techniques (Z score = 0.01) or management/research activities (Z score = −0.30). Most were early-career professionals (44.1% aged 26–34 years), highly educated (91.9% held bachelor’s degrees), yet predominantly held junior titles (45.9%). This role aligns with Benner’s novice/advanced beginner stage, requiring experience accumulation and pattern recognition through routine tasks. Despite 46.8% holding specialist certificates, their work remains confined to basic care—life support, medication, vital signs monitoring—primarily for patients with chronic infections such as HIV and HBV. Consistent with the novice transition phase, these nurses possess foundational skills but lack specialized expertise, necessitating gradual progression toward comprehensive or managerial roles (25). This distribution reflects the ladder-like development of infectious disease nursing roles. Critically, Type C nurses bore the heaviest weekly workload (82.0% ≥ 40 h/week) and exhibited high turnover intention (19.8%). This aligns with the Job Demands–Resources model (15). Stigmatization and psychological burden unique to infectious disease care compound this dilemma, with even basic tasks carrying perceived infection risk (26).

Thus, flexible scheduling and psychological support may reduce turnover and burnout while enhancing care quality and professional identity (27, 28). Additionally, recertification-linked specialist assessments could increase the use of advanced techniques among certificate holders. Selecting senior C-type nurses for development in clinical, teaching, and light managerial functions may facilitate transition to Type A roles, preventing role entrenchment and enhancing surge capacity during public health emergencies.

### 4.3 Influencing factors of infectious disease intensive care nurse classification

#### 4.3.1 Influence of age on type B and type C

When comparing management coordination (Type B) with clinical task execution (Type C), age was the only significant independent predictor. Nurses aged 26–34 and 35–45 were, respectively, 4.27 times (OR = 0.234, *p* = 0.041) and 4.17 times (OR = 0.240, *p* = 0.019) more likely to belong to Type C than Type B, relative to those >45 years. This reflects the typical engagement of early-career nurses in basic tasks (29) and highlights a structural misalignment: many mid-career nurses who actually perform teaching and coordination roles are not classified as Type B due to lacking formal management titles. This discrepancy underscores the fundamental difference between role identification based on administrative frameworks versus practice-based data. Nursing management systems should therefore establish more sensitive mechanisms to formally recognize and integrate nurses with demonstrated coordination potential into career pathways, enabling more precise talent pipeline development.

#### 4.3.2 Influence of age and nurse identity on type A and type B

When comparing management coordination (Type B) with comprehensive development (Type A), nurses aged 35–45 were more likely to belong to Type A than Type B (OR = 0.216, *p* = 0.009). This suggests that in China, comprehensive development serves as the primary pathway for mid-career professional advancement, whereas management coordination is more concentrated in late career. Nurses with multiple professional responsibilities (e.g., teaching, specialist, quality control) were 3.46 times more likely to be classified as Type A than Type B (OR = 0.289, *p* = 0.008), clearly distinguishing the formation paths of these two roles. Comprehensive development represents the horizontal deepening of clinical practice—nurses naturally extend derivative responsibilities such as teaching and quality control, forming competency composites consistent with Benner’s expert growth through diversified practice (30, 31). In contrast, management coordination involves vertical functional transformation, shifting focus from clinical operations to resource coordination and system management, which relies more on formal administrative authorization than on the natural extension of clinical roles. These findings suggest that cultivating comprehensive development nurses requires supporting the expansion of diverse professional responsibilities, while building management coordination capacity necessitates clear administrative promotion pathways and structured role transition mechanisms.

#### 4.3.3 No significant independent predictors between type A and type C

A key finding is that, after controlling for other variables, factors significant in univariate analyses—such as age, professional title, and registered nurse-only status—showed no statistically significant independent predictive effect in distinguishing comprehensive development (Type A) from clinical task execution (Type C). This does not negate the univariate results but deepens their interpretation.

First, it explains the non-significance of certain variables (e.g., gender, education, hospital type), likely due to the high homogeneity of China’s critical infectious disease nursing workforce—predominantly female, bachelor’s degree–educated, and concentrated in large public hospitals (32–34). Such homogeneity limits their discriminative power. The JD-R model further suggests that role differentiation stems more from specific configurations of job demands and resources and individual engagement than from static demographics (35, 36).

More importantly, the lack of significant demographic predictors validates our hypothesis: in this complex specialty, nurses’ functional roles are not linearly determined by age or title. This aligns with Benner’s theory that competence and role identity are developed and recognized through situated clinical practice (37–39). Whether a nurse becomes a comprehensive development type depends primarily on opportunities, demonstrated capabilities, and responsibilities undertaken in practice—a multifactorial, context-dependent process (40, 41). This underscores the strength of our practice-based clustering approach, which captures authentic functional differentiation invisible to traditional personnel systems.

In summary, while univariate and multivariate analyses together outline broad demographic profiles of nurse groups, traditional variables alone cannot explain or predict roles formed through complex practice. This reveals a gap: hospital personnel systems (seniority, title) inadequately reflect competency-based role differentiation. By clustering nurses based on actual work activities, this study provides an empirical foundation for transitioning from identity-based to role-based management.

## 5 Limitations and future directions

The research results have important theoretical and management implications, but there are also some limitations that need to be improved in future research.

### 5.1 Time window limitations

Data were collected from February to March 2025, a period reflecting routine respiratory infection seasons rather than major outbreak peaks. Thus, the identified role distributions, task participation patterns, and workload characteristics primarily represent normal operating conditions. During public health emergencies, nurse role boundaries, task combinations, and hierarchical structures may shift substantially due to surges in patient volume, workforce expansion, and duty reconfiguration. Generalizability to emergency contexts remains to be tested. Future research should employ multi-wave, cross-period surveys across influenza seasons, emerging outbreak phases, and emergency response windows to assess the stability and variability of this role typology under different epidemic scenarios.

### 5.2 Sample and external validity limitations

Convenience sampling resulted in a sample predominantly from tertiary public hospitals (81.5%), with underrepresentation of private and non-tertiary institutions. This limits extrapolation to primary care, community infectious disease units, and private specialty hospitals. Moreover, institutional variations in role design and task allocation across different facility sizes and resource levels could not be examined. The sample also exhibited high homogeneity in gender (95.6% female) and education (89.5% bachelor’s degree), partly explaining the non-significance of these variables in univariate analyses. Subsequent studies should adopt multi-stage stratified sampling to include secondary hospitals, community hospitals, and private infectious disease facilities, enabling comparisons across institutional tiers, geographic regions, and resource levels.

### 5.3 Measurement tool and content coverage limitations

Although the self-developed questionnaire demonstrated excellent reliability (Cronbach’s *α* = 0.965), its items were derived from literature review and nurse interviews reflecting current mainstream practices. Emerging responsibilities associated with novel pathogens, advanced technologies, and evolving outbreak response protocols may not be fully captured. Future research should establish a dynamically updated item bank aligned with pathogen trends, nursing innovation, and public health guidelines. Additionally, integrating patient-reported experience measures and multidisciplinary team feedback would enable a tripartite (nurse–physician–patient) evaluation framework for more comprehensive role effectiveness assessment.

### 5.4 Research design limitations

The cross-sectional design provides only a static snapshot of role distribution and cannot elucidate individual nurses’ role transition trajectories or the underlying drivers across career stages and epidemic cycles. Future longitudinal cohort studies are needed to track task participation patterns and role affiliations at multiple time points, capturing dynamic role evolution. Potential predictors at the individual level (e.g., educational advancement, research/teaching involvement, emergency deployment experience, psychological resilience), organizational level (e.g., structural empowerment, supervisory support), and system level (e.g., policy incentives) should be incorporated to model transition pathways.

As an exploratory study, this work identified three functional nursing roles in critical infectious disease care and demonstrated the limited predictive power of traditional demographic and professional variables. Future research should extend along temporal, contextual, variable, and methodological dimensions to generate robust evidence supporting the institutional shift from identity-based to role-based management.

## 6 Conclusion

Based on Benner theory and JD-R model, this study used K-means cluster analysis to identify three types of practice driven roles in 275 critically ill infectious disease nursing nurses in multiple centers: comprehensive development type, management coordination type and clinical task execution type. The three types of roles present clear functional differentiation: the comprehensive development type is the horizontal deepening of clinical practice, forming the ability complex in the derivative duties of teaching, quality control, etc.; The management coordination type is the vertical transformation of functions, from direct care to system coordination; Clinical task execution is the basic platform for the transition from novice to competent, and it undertakes the most onerous front-line care work. Multiple regression showed that the independent predictive ability of traditional variables such as age and professional title to role attribution was extremely limited. This finding verifies that the functional role of nurses in infectious disease intensive care is not linearly determined by static identity tags, but gradually formed in the practice of coping with specific clinical situations. This study provides a reference for the transformation of nurses from identity management to role management.

## Data Availability

The original contributions presented in the study are included in the article/supplementary material, further inquiries can be directed to the corresponding authors.
